# MEK/ERK-Mediated Transcriptional Repression of Metabolic Genes Confers Host Defense Against *Gallid alphaherpesvirus 1* Infection via Modulating Fos Nuclear Translocation

**DOI:** 10.3390/microorganisms14071492

**Published:** 2026-07-08

**Authors:** Lu Cui, Yu Zhang, Haixia Zhang, Shufeng Feng, Yongxin Zhu, Xuefeng Li, Pengfei Liu, Shengwang Liu, Hai Li

**Affiliations:** 1 National & Local Joint Engineering Research Center of Biodiagnosis and Biotherapy, The Second Affiliated Hospital of Xi’an Jiaotong University, Xi’an 710061, China; cuilu@xjtu.edu.cn (L.C.); lixuefeng@xjtu.edu.cn (X.L.); liupengfei@xjtu.edu.cn (P.L.); 2 School of Basic Medical Sciences, Xi’an Jiaotong University Health Science Center, Xi’an Jiaotong University, Xi’an 710061, China; zhangyu2022@xjtu.edu.cn (Y.Z.); zhanghaixia@stu.xjtu.edu.cn (H.Z.); feng_xjtu0204@163.com (S.F.); zyx2023@stu.xjtu.edu.cn (Y.Z.); 3 Division of Avian Infectious Diseases, State Key Laboratory of Veterinary Biotechnology, National Poultry Laboratory Animal Resource Center, Harbin Veterinary Research Institute, The Chinese Academy of Agricultural Sciences, Harbin 150069, China; 4 Translational Medicine Institute, Xi’an Jiaotong University Health Science Center, Xi’an 710061, China; 5 MOE Key Laboratory of Environment and Genes Related to Diseases, Xi’an Jiaotong University, Xi’an 710061, China; 6 Key Laboratory for ImmunoHealth of Shaanxi Province, Xi’an 710061, China

**Keywords:** alphaherpesvirus, MEK/ERK signaling pathway, Fos, nuclear translocation, cellular metabolic pathways, *Gallid alphaherpesvirus 1*

## Abstract

Infectious laryngotracheitis virus (ILTV), formally known as *Gallid alphaherpesvirus 1*, represents a prominent alphaherpesvirus that poses a significant threat to the global poultry industry. Current routine vaccination strategies fail to eliminate latent infection. Host–pathogen interaction networks have become a key focus in antiviral research. Our previous study demonstrated that activation of the MEK/ERK signaling pathway upon ILTV infection restricts host cellular metabolic activity to mount protective host antiviral responses, yet the underlying molecular mechanism remains unclear. The present work systematically dissects the contribution of MEK/ERK signaling to intrinsic host defense against ILTV. The results show that ILTV infection activates the MEK/ERK pathway, which in turn promotes the expression, activation, and nuclear translocation of the transcription factor Fos. As a core transcriptional regulator, Fos represses host metabolic gene expression, thereby restricting viral replication and proliferation. Integrated multi-omics analyses further demonstrate that the MEK/ERK-Fos-metabolic regulatory axis operates uniformly during infection with avian, human, and porcine alphaherpesviruses, suggesting a broadly conserved host antiviral mechanism. This consistent cross species signaling pattern points toward an evolutionarily conserved host antiviral strategy and provides potential molecular targets for the development of broad-spectrum antiviral strategies against alphaherpesviruses.

## 1. Introduction

The subfamily Alphaherpesvirinae of the family Herpesviridae comprises double-stranded DNA viruses characterized by broad host ranges, diverse cell tropisms, and the ability to establish latent infection. Its members include important pathogens endangering livestock and poultry farming, such as avian *Gallid alphaherpesvirus 1* (infectious laryngotracheitis virus, ILTV) [1], Marek’s disease virus (MDV) [2], and porcine pseudorabies virus (PRV) [3], as well as human pathogens that seriously threaten public health, including herpes simplex virus type 1 (HSV-1) [4,5] and varicella-zoster virus (VZV) [6,7]. Among them, ILTV causes acute and highly contagious respiratory diseases in chickens, leading to decreased egg production and increased mortality, which continuously causes significant economic losses to the global poultry industry. Meanwhile, ILTV can establish lifelong latent infection in the chicken trigeminal ganglion [8]. Current vaccines can only prevent clinical onset but fail to completely eliminate the virus from the host, and effective therapeutic drugs are lacking [9]. Similar to other alphaherpesviruses, ILTV replication and latency establishment are highly dependent on the reprogramming of host cell signal transduction, transcriptional regulation, and metabolic networks [1]. Therefore, dissecting the key regulatory mechanisms of virus–host interactions provides a theoretical basis for the development of novel antiviral intervention strategies.

The mitogen-activated protein kinase (MAPK) pathway is the core cascade pathway mediating extracellular signal transmission to the nucleus in eukaryotic cells. It mainly includes three classical branches, namely MEK/ERK, p38 MAPK, and JNK, which play critical roles in cell proliferation, apoptosis, metabolic regulation, and innate immunity [10,11]. Accumulating evidence has confirmed that the MAPK pathway is widely involved in the interaction between various herpesviruses and their hosts, and is a key node affecting viral replication and host antiviral responses [7,12,13,14]. Our previous studies found that ILTV infection can biphasically activate host MEK/ERK and p38 MAPK cascade pathways [15]. Among them, the MEK/ERK pathway is specifically activated at the early stage of infection and exerts antiviral effects by inhibiting host cell metabolism. However, its underlying molecular mechanism remains unclear. Fos, a core transcription factor of the activator protein 1 (AP-1) transcription complex and a canonical downstream effector of the MEK/ERK pathway, has been demonstrated in our previous studies to participate in ILTV infection and modulate viral replication [16,17]. However, the specific signaling pathway regulating Fos expression and activation during ILTV infection remains to be systematically investigated.

Therefore, using ILTV-infected chicken hepatoma (LMH) cells as the experimental model, we systematically elucidated the molecular mechanism by which the MEK/ERK pathway modulates the expression of host metabolic genes to constrain ILTV infection. This study provides a mechanistic basis for understanding alphaherpesvirus-host interplay and offers promising therapeutic targets for the development of broad-spectrum anti-herpesviral strategies.

## 2. Materials and Methods

### 2.1. Viral Strain and Cells

We used the virulent ILTV-LJS09 strain (GenBank Accession No. JX458822) for all infection assays in this study, which is preserved at the Harbin Veterinary Research Institute of the Chinese Academy of Agricultural Sciences (CAAS). We propagated this strain in the chemically immortalized leghorn male hepatoma (LMH) cell line, which develops clear and consistent cytopathic effects (CPEs) during the infection cycle [18,19]. We maintained the LMH cell line (ATCC CRL-2117) in Dulbecco’s modified Eagle’s medium (DMEM) supplemented with 10% fetal bovine serum (FBS), 100 units/mL penicillin, 100 μg/mL streptomycin, and 2 mM L-glutamine. All cell cultures were incubated in a humidified 37 °C incubator with 5% CO_2_.

### 2.2. Viral Growth Assays

We performed single-infection assays and one-step growth curves of ILTV in LMH cells. For these assays, we seeded cells into 24-well plates and cultured them overnight until reaching 80–90% confluence. The next morning, we replaced the original culture medium with fresh medium containing either dimethyl sulfoxide (DMSO, vehicle control) or MEK/ERK inhibitors at the indicated working concentrations. After 2 h of pretreatment, we inoculated cells with ILTV at a multiplicity of infection (MOI) of 0.01, with fresh inhibitors maintained in the medium throughout the entire infection process. Two highly selective MEK1/2 inhibitors were used in this study, with the following working concentrations: Binimetinib (MCE, HY-15202, Monmouth Junction, NJ, USA) at 1 μM, and PD0325901 (MCE, HY-10254, Monmouth Junction, NJ, USA) at 0.25 μM. As validated in our prior work, both inhibitors at the above working concentrations significantly suppress MEK/ERK pathway activation without inducing obvious cytotoxicity or impairing normal cell growth [15]. We collected aliquots of culture supernatant at predefined time points post-infection, and quantified viral replication levels via 50% tissue culture infective dose (TCID_50_) analysis and ILTV-specific real-time quantitative PCR (qPCR), as detailed in our previous publication [20].

### 2.3. Western Blotting Analysis

We performed western blotting assays strictly following the protocol established in our previous work, with only minor optimizations to washing conditions [21]. Briefly, we rinsed harvested cells with ice-cold PBS, then extracted soluble total proteins using Strong RIPA Lysis Buffer (Beyotime Biotech, P0013B, Shanghai, China) supplemented with Phosphatase Inhibitor Cocktail II (Abcam, ab201113, Cambridge, MA, USA). We measured the protein concentration of each sample with a BCA assay kit (Beyotime Biotech, P0009, Shanghai, China). After denaturation, we loaded equal amounts of protein for sodium dodecyl sulfate-polyacrylamide gel electrophoresis (SDS-PAGE) separation, then transferred the resolved proteins onto nitrocellulose (NC) membranes (Millipore, HATF00010, Billerica, MA, USA) or polyvinylidene fluoride (PVDF) membranes (Sigma-Aldrich, IPVH00010, St. Louis, MO, USA). We blocked the membranes with 5% non-fat milk for 1 h at room temperature, then incubated them overnight at 4 °C with the following primary antibodies: phospho-MEK1/2 (Abcam, ab4750, Cambridge, MA, USA), total MEK1/2 (CST, 9122, Danvers, MA, USA), phospho-ERK1/2 (Abmart, T40072F, Shanghai, China), total ERK1/2 (Abmart, T40071, Shanghai, China), p-Fos (Ser362) (Abmart, TA3053S, Shanghai, China), total Fos (Abmart, TA0132S, Shanghai, China), p-JUN (Abmart, PN340810S, Shanghai, China), total JUN (Abmart, PA1634S, Shanghai, China), HSP90 (Abmart, PA1558S, Shanghai, China), Lamin B (Abmart, JQ028291S, Shanghai, China), HA tag antibody (Beyotime Biotech, AF2858, Shanghai, China) and tubulin (Sigma, T6119, St. Louis, MO, USA). The next day, we washed the membranes three times with TBST (10 min per wash), then incubated them with matched fluorescent secondary antibodies for 1 h at room temperature. After three additional 10-min TBST washes, we visualized protein band signals using an Odyssey CLX infrared imaging system (LiCor Biosciences, Lincoln, NE, USA).

### 2.4. Nuclear and Cytoplasmic Fractionation Assay

To separate cytoplasmic and nuclear protein fractions from each experimental group, a commercial nuclear-cytoplasmic protein extraction kit (Beyotime Biotech, P0028, Shanghai, China) was used, with all operations performed strictly in accordance with the manufacturer’s protocol. Briefly, harvested cells were first rinsed twice with ice-cold PBS, then resuspended in freshly prepared cytoplasmic extraction buffer and incubated on ice for 15 min with gentle mixing every 5 min. Following centrifugation at 2000× *g* for 5 min at 4 °C, the clear supernatant was carefully collected as the cytoplasmic protein fraction. The remaining nuclear pellet was washed twice with pre-chilled PBS to remove residual cytoplasmic contaminants, resuspended in nuclear protein lysis buffer supplemented with protease and phosphatase inhibitors, and incubated on ice for 30 min with intermittent vortexing. After a final centrifugation step at 12,000× *g* for 15 min at 4 °C, the supernatant containing solubilized nuclear proteins was transferred to a new pre-cooled tube. Protein concentrations of both cytoplasmic and nuclear fractions were determined using the BCA assay. Subsequent Western blot analysis was carried out as described in Section (Western blotting analysis), with HSP90 serving as the loading control for cytoplasmic proteins and Lamin B as the specific marker for nuclear proteins.

### 2.5. Immunofluorescence Staining

For immunofluorescence assays, we seeded LMH cells in 35-mm glass-bottom cell culture dishes for confocal imaging, then infected the cells with ILTV at an MOI of 1. At 20 h post-infection, we rinsed both infected (experimental group) and mock-infected (control group) samples with PBS, then fixed the cells with 4% paraformaldehyde for 30 min. We quenched excess aldehyde groups, permeabilized cells with 0.1% Triton X-100, and blocked non-specific antibody binding with 2% bovine serum albumin (BSA) for 1 h at room temperature. We then incubated the samples with rabbit polyclonal antibody against p-Fos (Ser362) overnight at 4 °C, followed by a 1-h incubation with FITC-conjugated goat anti-rabbit secondary antibody (Jackson Laboratory, Bar Harbor, ME, USA) at room temperature. We counterstained all cell nuclei with 4′,6-diamidino-2-phenylindole (DAPI), and captured fluorescence images using an LSM880 confocal microscope system (Zeiss, Oberkochen, Germany).

### 2.6. Plasmid Construction and Cell Transfection

The *p*CAG-HA expression vector, which carries an N-terminal HA tag, was constructed as previously description [22]. To generate the Fos overexpression construct, the full-length coding sequence of chicken Fos was amplified from LMH cell cDNA, with corresponding primer sequences listed in Table 1. All PCR reactions were carried out using KOD-Plus-Neo high-fidelity DNA polymerase (TOYOBO, KOD-401, Osaka, Japan) to ensure amplification accuracy. Following amplification, the PCR products were purified and subjected to double restriction enzyme digestion with *Xho*I and *Kpn*I. The digested target fragment was then gel-purified and ligated into the linearized *p*CAG-HA vector using T4 DNA ligase (NEB, M0202, Ipswich, MA, USA) to generate the recombinant *p*CAG-Fos-HA plasmid. All recombinant constructs were verified by Sanger sequencing to confirm correct insertion orientation and absence of unintended mutations. For transient transfection assays, LMH cells were seeded into tissue culture plates 12 h prior to transfection. Transfections were performed using Turbofect transfection reagent (Thermo Scientific, R0531, Waltham, MA, USA) strictly following the manufacturer’s recommended protocol.

### 2.7. Quantitative Reverse Transcription PCR (RT-qPCR)

We isolated total RNA from harvested cells using the EasyPure RNA Purification Kit (TransGen Biotech, ER101, Beijing, China) strictly following the manufacturer’s protocol. We performed both relative and absolute RT-qPCR using the SYBR PrimeScript^TM^ Kit (TaKaRa Bio Inc., RR047A, Tokyo, Japan), as described in our previous work [21]. For relative gene expression quantification, we calculated the data with the 2^−ΔΔCT^ method, and presented the results as Log_2_ fold change or fold change relative to the control group. For absolute RT-qPCR, we prepared standard curves by cloning the PCR product of the ILTV *gC* gene into the *p*MD18-T plasmid (TaKaRa Bio Inc., 6011, Tokyo, Japan) following the manufacturer’s instructions. Primer sequences are presented in Table 1, and all reactions were performed in technical triplicate.

### 2.8. Chromatin Immunoprecipitation (ChIP) Assays

We carried out ChIP experiments following a previously published method with minor modifications [23]. Briefly, we fixed LMH cells with 1% formaldehyde for 10 min at room temperature, then terminated the crosslinking reaction with 0.125 M glycine. We sheared crosslinked genomic DNA into 200–500 bp fragments using a 6-mm probe sonicator (Cole Parmer, CPX500, Vernon Hills, IL, USA), with samples kept on ice throughout the process to prevent overheating. The sonication program consisted of 20 cycles (30 s pulse/30 s rest) at an amplitude of 30%. For each ChIP reaction, we used sheared chromatin prepared from 5 × 10^6^ LMH cells, and incubated the chromatin with 5 μg of anti-HA antibody or isotype control IgG overnight at 4 °C. We performed pull-down of antibody-bound chromatin complexes using Protein A/G PLUS-agarose beads, following the manufacturer’s instructions (Santa Cruz Biotechnology, sc-2003, Dallas, TX, USA). We purified the immunoprecipitated DNA with a QIAquick PCR Purification Kit (QIAGEN, 28106, Hilden, Germany). We performed ChIP coupled with quantitative PCR (ChIP-qPCR) using Luna Universal qPCR Master Mix (NEB, M3003L, Ipswich, MA, USA) on a Bio-Rad CFX96 (Hercules, CA, USA) instrument, following the manufacturer’s protocol. Primer sequences are provided in Table 1, and we ran all samples in technical triplicate.

### 2.9. RNA Interference

We used sequence-specific short interfering RNAs (siRNAs) to knock down endogenous Fos expression in LMH cells, with all siRNA oligonucleotides synthesized by Sigma. The siRNA targeting chicken Fos mRNA (NM_205508; termed siFos) has the sequence 5′-CCGACACUCUGCAGGCGGA-3′; a non-targeting siRNA with no homologous sequence in the chicken genome (termed siControl, sequence 5′-UUCUCCGAACGUGUCACGUTT-3′) was used as a negative control. For siRNA transfection, we seeded subconfluent LMH cells into 24-well plates, then transfected 5 pmol of pooled siRNAs into the cells using Lipofectamine RNAiMAX (Invitrogen, 13778075, Waltham, MA, USA) following the manufacturer’s protocol. At 24 h post-transfection, we infected the siRNA-transfected cells with ILTV at an MOI of 1. We harvested total RNA from the cells at 12 h post-infection for subsequent gene expression analysis.

### 2.10. RNA Sequencing

For genome-wide transcriptome profiling, we collected ILTV-infected LMH cells from three treatment groups: DMSO control group, Binimetinib (BI) inhibitor group, and PD0325901 (PD) inhibitor group. Each group included three independent biological replicates. We extracted total RNA from harvested cell samples using the RNeasy Plus Mini Kit (QIAGEN, 74134, Hilden, Germany) following the manufacturer’s guidelines. We assessed the integrity, purity and concentration of all RNA samples via agarose gel electrophoresis and analysis (Agilent Technologies, G2939A, Santa Clara, CA, USA), and only samples that passed quality control were used for downstream experiments. We constructed mRNA sequencing libraries using the Illumina standard library preparation kit (Illumina, Inc., 20020594, San Diego, CA, USA) strictly according to the manufacturer’s protocol. All qualified libraries were finally sequenced on the Illumina NovaSeq platform by BGI Genomics Co., Ltd., Wuhan, China.

### 2.11. High-Throughput Data Analysis

We processed and analyzed raw RNA sequencing data with the Galaxy web-based analysis platform [24]. Raw reads were processed on the Galaxy platform through quality control, genome alignment, gene quantification and differential expression analysis. FastQC (v0.11.9) and Trimmomatic (v0.36.5) were used for quality filtering, with a Q30 base ratio greater than 90%. HISAT2 (v2.2.1), featureCounts (v2.0.1) and DESeq2 (v1.34.0) were adopted for subsequent analyses, and genes with |log_2_(Fold Change)| ≥ 1 and adjusted *p*-value less than 0.05 were defined as differentially expressed genes. We performed pathway enrichment analysis using the DAVID tool (https://davidbioinformatics.nih.gov/home.jsp, accessed on 12 November 2025), with the EASE Score (a modified Fisher exact *p-*value) set as the significance threshold for gene enrichment [25]. All raw RNA-seq data generated in the current study have been deposited in NCBI BioProject under accession number PRJNA1079397.

### 2.12. Public Dataset Acquisition and Conservation Analysis

For cross-virus conservation analysis of alphaherpesvirus-induced transcriptional responses, transcriptomic datasets from diverse alphaherpesvirus infection systems were retrieved from the NCBI Gene Expression Omnibus (GEO) database (https://www.ncbi.nlm.nih.gov/geo/, accessed on 31 December 2025), covering the following models: Marek’s disease virus (MDV) infection, pseudorabies virus (PRV) infected porcine testicular (ST) cells and murine microglial (BV2) cells, herpes simplex virus type 1 (HSV-1) infected human lung adenocarcinoma (A549) cells and human foreskin fibroblasts (HFF), as well as varicella-zoster virus (VZV) infected human neuroblastoma cells (SH-SY5Y). The corresponding GEO accession numbers of these datasets are GSE124133 [26], GSE201012 [27], GSE247533 [28], GSE237079 [29], GSE314009 [30], and GSE141932 [6], respectively. All downloaded datasets were processed using the same standardized analytical pipeline described earlier to ensure comparability of results. This pipeline included differential expression gene screening and Kyoto Encyclopedia of Genes and Genomes (KEGG) pathway enrichment analysis for each individual dataset [31]. To identify evolutionarily conserved regulatory pathways activated during alphaherpesvirus infection, an intersection plot of significantly enriched pathways across all infection models was generated using Upset analysis.

### 2.13. Transcription Factor Prediction and Interaction Network Construction

We constructed a protein–protein interaction (PPI) network for differentially expressed metabolism-related genes using the STRING database (https://string-db.org/, accessed on 12 November 2025), and predicted upstream transcription factors targeting these genes through the ChEA3 database (https://maayanlab.cloud/chea3/, accessed on 12 November 2025). We visualized the interaction network and screened core hub transcription factors using Cytoscape software (v3.9.1). Additionally, we identified conserved core transcriptional regulators across different alphaherpesvirus infection systems via Upset analysis.

### 2.14. Multiple Sequence Alignment and Protein 3D Structure Homology Modeling

To investigate the evolutionary conservation of Fos protein structure and function across species, full-length amino acid sequences of Fos from four representative species were retrieved from the NCBI Protein Database (https://www.ncbi.nlm.nih.gov/protein/, accessed on 12 November 2025): chicken (*Gallus gallus*, NP_990631.1), mouse (*Mus musculus*, NP_034364.1), human (*Homo sapiens*, NP_005243.2), and pig (*Sus scrofa*, NP_999174.1). Kinase target prediction and phosphorylation site analysis were performed using the online GPS 6.0 server (https://gps.biocuckoo.cn/online.php, accessed on 12 November 2025), with MAPK family kinases selected as the target kinase group. Multiple sequence homology alignment was conducted using the online MUSCLE tool from EMBL-EBI (https://www.ebi.ac.uk/jdispatcher/msa/muscle, accessed on 12 November 2025) to analyze the sequence conservation of Fos proteins, with a specific focus on the core region containing the predicted conserved phosphorylation sites. Full-length three-dimensional structure homology models of Fos proteins from the four species were generated using AlphaFold3, and the highest-confidence models were selected for further analysis. All protein structures were visualized using PyMOLTM Molecular Graphics System, Version 2.6.0a0 (Schrödinger, New York, NY, USA) [32].

### 2.15. Statistical Analysis

The SPSS software package (SPSS for Windows version 13.0, SPSS Inc., Chicago, IL, USA) was used for all statistical analyses. Data obtained from several experiments are reported as the mean ± standard deviation (SD). The significance of differences between two groups was determined with two-tailed Student’s *t*-test. One-way or two-way analysis of variances with Bonferroni correction was employed for multi-group comparison. For all analyses, a probability (*p*) value of <0.05 was considered statistically significant.

## 3. Results

### 3.1. MEK/ERK Pathway Regulates Key Host Metabolic Pathways During ILTV Infection

**Figure 1:**
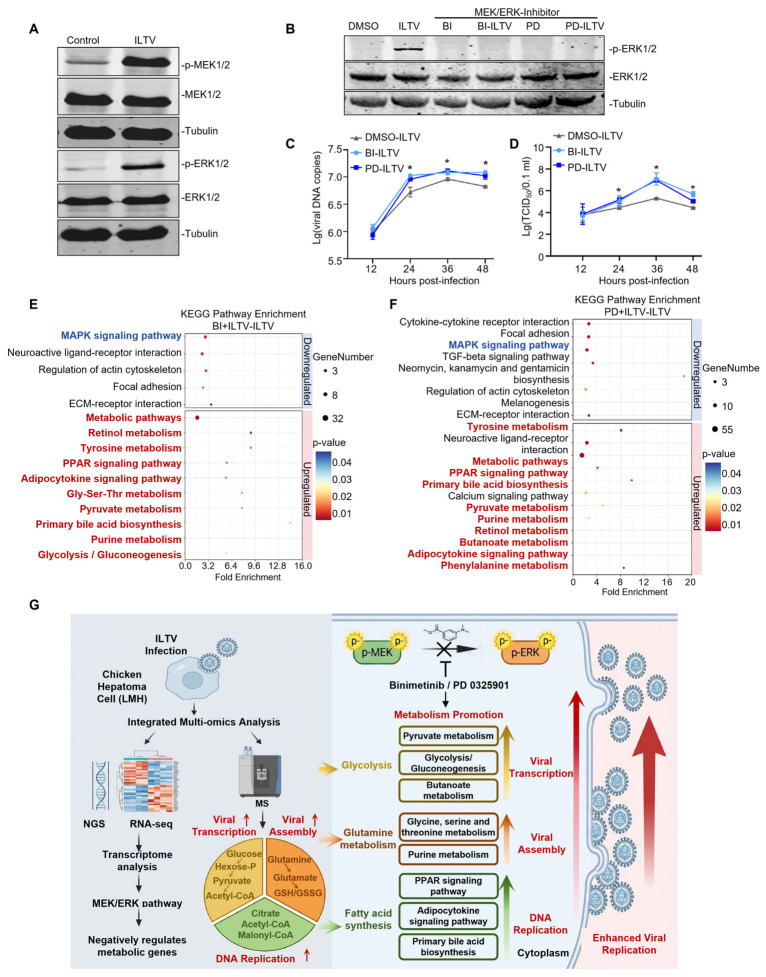
Activation of the MEK/ERK pathway and its regulatory effects on ILTV replication and host metabolic pathways. (**A**) Western blot analysis of phosphorylated ERK (p-ERK) and total ERK protein expression levels in cells after ILTV infection. (**B**) Western blot analysis of p-ERK and total ERK protein expression levels in ILTV-infected cells after treatment with MEK/ERK inhibitors. Real-time quantitative PCR (RT-qPCR) and TCID_50_ assay were used to detect viral genome copy number (**C**) and infectious viral titer (**D**) in ILTV-infected cells after MEK/ERK inhibitor treatment, respectively. KEGG pathway enrichment bubble plots of differentially expressed genes in LMH cells after treatment with the MEK/ERK inhibitors BI (**E**) and PD (**F**). The MAPK signaling pathway significantly inhibited by both inhibitors is labeled in blue font. The metabolism-related pathways significantly upregulated by both inhibitors are labeled in red font. Bubble size represents the number of differentially expressed genes enriched in the pathway. Bubble color represents the significance *p*-value of pathway enrichment. (**G**) Integrated analysis of transcriptome and metabolomics data, which illustrates the key metabolic modules regulated by the MEK/ERK pathway during ILTV infection and their corresponding relationships with viral replication stages: glycolysis/glucose metabolism is mainly involved in viral early transcription, glutamine metabolism mainly supports early gene expression and nucleotide synthesis, while fatty acid metabolism is mainly involved in viral particle assembly and release. After MEK/ERK pathway inhibition, the above metabolism-related pathways are overall restored and upregulated, suggesting that this pathway exerts anti-ILTV infection effects by inhibiting metabolic programs dependent on viral replication. The results in (**C**,**D**) are presented as mean ± SD, *n* = 3. Asterisks indicate statistical difference (*p* < 0.05).

To clarify the role of the MEK/ERK pathway during ILTV infection and its downstream molecular mechanisms governing host metabolic reprogramming and viral replication, we selected LMH cells, currently the only permissive cell line supporting stable in vitro ILTV infection, as our in vitro experimental model, consistent with our previous work validating this cell line as an ideal platform for investigating ILTV-host cell interactions [15,17,20,33,34,35]. Based on this, we first detected the activation status of the host MEK/ERK pathway after ILTV infection by Western blot. The results showed that ILTV infection significantly upregulated the phosphorylation levels of MEK1/2 and ERK1/2, while the total protein expression of MEK1/2 and ERK1/2 did not change significantly, confirming that ILTV infection can specifically activate the host MEK/ERK signaling pathway (Figure 1A). Western blot validation showed that both inhibitor treatments significantly inhibited ERK1/2 phosphorylation induced by ILTV infection without affecting total ERK1/2 protein expression, achieving specific blockade of the MEK/ERK pathway (Figure 1B). Subsequently, we detected the effect of MEK/ERK pathway blockade on ILTV replication by real-time quantitative PCR (RT-qPCR) and TCID_50_ assay, respectively. The results showed that specific inhibition of the MEK/ERK pathway led to significant upregulation of both ILTV viral genome copy number and infectious viral titer, suggesting that activation of the MEK/ERK pathway plays a host antiviral defense role during ILTV infection (Figure 1C,D).

To further analyze the characteristics of transcriptional changes in host metabolism affected by the MEK/ERK pathway during ILTV infection, we performed whole-genome transcriptome sequencing and KEGG pathway enrichment analysis in cells infected with ILTV that had been treated with the MEK/ERK inhibitors BI and PD. The results showed that after MEK/ERK pathway inhibition, the expression of genes related to the MAPK signaling pathway was significantly downregulated, while multiple core metabolism-related pathways such as host glycolysis/gluconeogenesis, lipid metabolism, amino acid metabolism, pyruvate metabolism, and PPAR signaling pathway were significantly enriched and upregulated (Figure 1E,F). These findings suggest that the antiviral function of the MEK/ERK pathway is closely related to its negative transcriptional regulation of the host metabolic network. To further determine whether the metabolism-related pathways restored and upregulated after MEK/ERK pathway inhibition are consistent with the metabolic programs dependent on ILTV replication, we compared the transcriptome results of this study with our previous metabolomics analysis of ILTV infection. The glycolysis/glucose metabolism, glutamine metabolism, and fatty acid metabolism, which are required for viral early transcription, nucleotide synthesis, and viral assembly and release, respectively, during ILTV replication, were analyzed [36]. After MEK/ERK pathway inhibition, these metabolic pathways related to ILTV replication showed a significant overall upregulation trend (Figure 1G). Collectively, these data reveal that the metabolic modules downregulated by the MEK/ERK pathway during ILTV infection are highly consistent with the core metabolic networks required for viral replication, suggesting that the MEK/ERK pathway may restrict ILTV replication by continuously inhibiting these metabolic programs.

### 3.2. MEK/ERK Promotes Fos Expression and Activation Through Both Transcriptional Regulation and Post-Translational Modification

**Figure 2:**
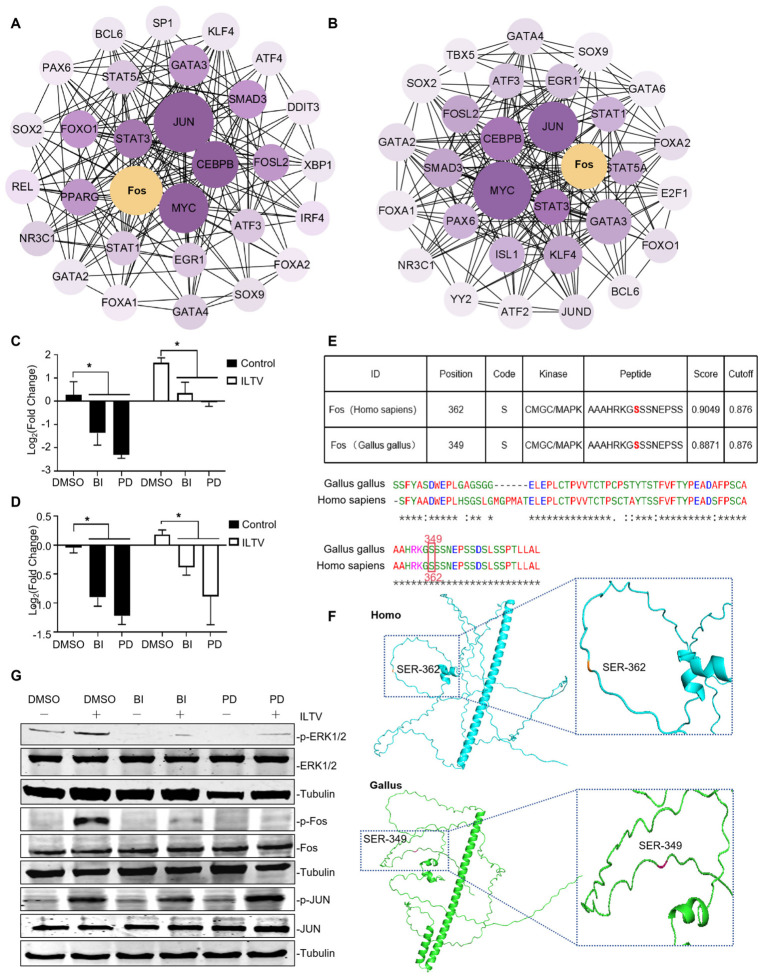
MEK/ERK promotes Fos expression and activation through both transcriptional regulation and post-translational modification (**A**,**B**) Prediction of upstream transcriptional regulators and construction of protein interaction networks for differentially expressed genes in metabolism-related pathways following treatment with BI (**A**) and PD (**B**) inhibitors. Node size is positively correlated with the network centrality (Degree value) of the gene. Node color from dark purple to light purple represents centrality from high to low. (**C**,**D**) RT-qPCR detection of *Fos* (**C**) and *JUN* (**D**) mRNA transcription levels after treatment with MEK/ERK inhibitors Binimetinib (BI) and PD0325901 (PD). (**E**) Homology alignment and kinase prediction analysis of Fos protein phosphorylation sites between chicken (*Gallus gallus*) and human (*Homo sapiens*). The upper panel shows phosphorylation sites predicted by GPS 6.0, with human S362 and chicken S349 being high-confidence phosphorylation sites. The lower panel shows the results of MUSCLE multiple sequence alignment. The local short sequence containing S362/S349 is completely conserved between human and chicken species. The conserved phosphorylated serine (S) site is labeled in red. * indicates completely conserved amino acid residues. (**F**) Three-dimensional structural models of human and chicken Fos predicted by AlphaFold3 and visualized with PyMOL. Ser362 and Ser349 are located within the intrinsically disordered region and fully exposed on the protein surface. The left panel shows the full-length three-dimensional structure of Fos. The right panel provides an enlarged view of the region containing the phosphorylation site, highlighting the spatial positions of human Fos Ser362 and chicken Fos Ser349. (**G**) Western blot analysis of the effects of MEK/ERK inhibitor BI and PD treatment on total and phosphorylated protein levels of ERK1/2, Fos and JUN in uninfected cells and cells exposed to ILTV. Tubulin was used as a protein loading control. + and − indicate ILTV infection and mock treatment, respectively. Data in (**C**,**D**) are presented as mean ± SD, *n* = 3. Asterisks indicate statistical significance (*p* < 0.05).

To further screen upstream regulators of metabolic pathways, we used the STRING database combined with the ChEA3 tool to predict upstream transcription factors of differentially expressed metabolic genes and construct a protein–protein interaction (PPI) network. Network analysis showed that AP-1 family transcription factors Fos and JUN had high connectivity in this transcriptional regulatory network, while transcription factors such as STAT3, MYC, CEBPB, and ATF3 also had high network centrality, suggesting that they may be involved in the regulation of host metabolic gene expression (Figure 2A,B). Based on the above transcriptome sequencing and bioinformatics analysis results, AP-1 family transcription factors Fos and JUN were initially identified as core candidate transcription factors downstream of the MEK/ERK pathway regulating host metabolic pathways. Integrating findings from our previous work, we confirmed that Fos can not only directly bind to the promoter region of the ILTV immediate early gene *ICP4* to regulate viral transcription, but also reshape the cellular metabolic network by transcriptionally regulating host metabolism-related genes [17]. Given that Fos and JUN belong to the AP-1 transcription factor family, we performed systematic molecular biological validation to further analyze the regulatory characteristics of the MEK/ERK pathway on Fos and JUN. To verify the direct regulatory effect of the MEK/ERK pathway on *Fos* and *JUN* transcription levels, we used MEK/ERK inhibitors Binimetinib (BI) and PD0325901 (PD) to target host cells and detected their mRNA transcription levels by RT-qPCR. The results showed that specific blockade of the MEK/ERK pathway significantly downregulated the mRNA transcription levels of both *Fos* and *JUN* in both uninfected (Control) and ILTV-infected cells (Figure 2C,D), confirming that the MEK/ERK pathway is the key upstream signaling pathway regulating *Fos* and *JUN* gene transcription in host cells. Since no specific commercial antibody is available to detect phosphorylated Fos in avian samples, we performed kinase target prediction and homology alignment analysis. The results showed that both human Fos Ser362 and chicken Fos Ser349 are high-confidence targets of MAPK family kinases, with prediction scores higher than the set threshold (Figure 2E, upper panel). The core region containing this serine phosphorylation site is completely conserved between humans and chickens, with 100% identity of the corresponding amino acid residues (Figure 2E, lower panel), confirming that human anti-phospho-Fos (Ser362) antibody is suitable for detecting phosphorylated Fos in chicken samples.

To further clarify the spatial structural characteristics of this conserved phosphorylation site, we performed three-dimensional structure homology modeling of human and chicken Fos proteins using AlphaFold3, respectively, and completed structural visualization and site spatial localization using PyMOL software. Three-dimensional structure analysis showed that both human Fos Ser362 and chicken Fos Ser349 sites are not embedded in the α-helix core structure of the protein, but are completely exposed in the intrinsically disordered region (IDR) of the protein. This spatial conformation feature provides sufficient structural accessibility for phosphorylation modification of this site by upstream kinases (Figure 2F). On this basis, we further verified the regulatory effect of the MEK/ERK pathway on Fos and JUN phosphorylation modification by Western blot. The results showed that ILTV infection significantly induced upregulation of Fos phosphorylation level (p-Fos) in host cells, while MEK/ERK inhibitor treatment significantly reversed ILTV infection-induced Fos phosphorylation activation. In contrast, MEK/ERK pathway blockade had no significant regulatory effect on the phosphorylation level (p-JUN) of another core transcription factor JUN in the metabolic regulatory network (Figure 2G), confirming that the MEK/ERK pathway has specific regulatory effects on Fos transcriptional activation and phosphorylation modification.

Taken together, these observations identify Fos as a critical MEK/ERK downstream transcription factor governing host metabolic homeostasis. The MEK/ERK pathway can synergistically regulate Fos activation levels at both transcriptional and post-translational modification levels, laying a theoretical foundation for subsequent dissection of the molecular mechanism by which Fos mediates host metabolic gene expression and thus ILTV infection.

### 3.3. MEK/ERK Pathway Promotes Fos Nuclear Translocation

**Figure 3:**
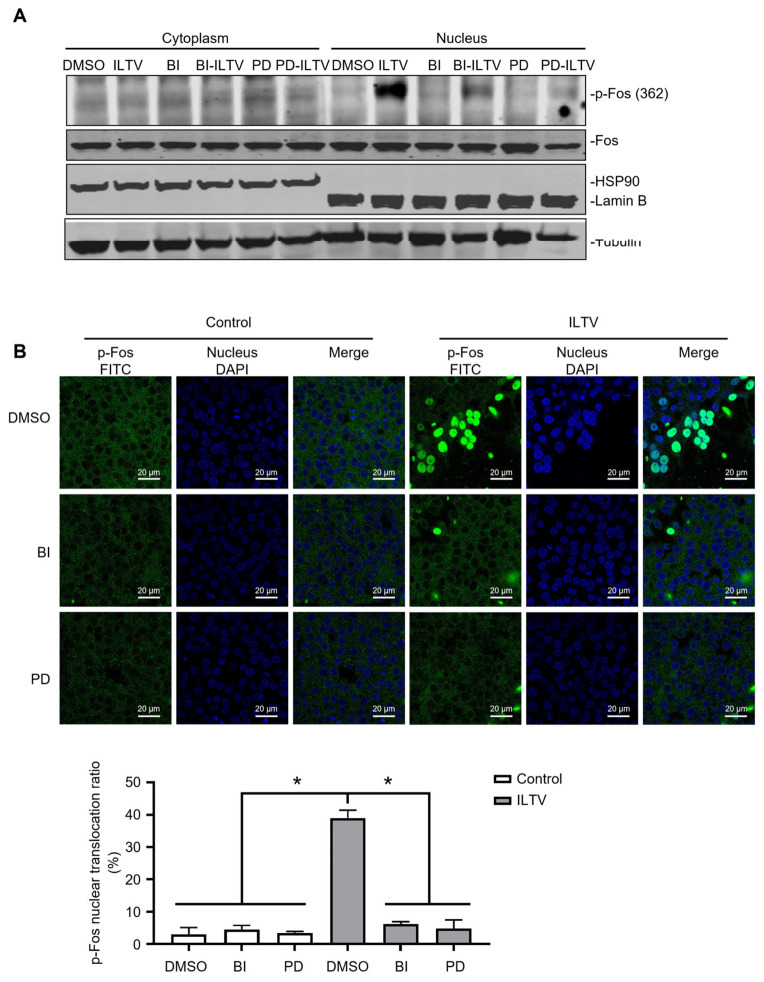
The MEK/ERK pathway regulates Fos subcellular localization by promoting Fos phosphorylation (**A**) Nuclear-cytoplasmic fractionation combined with Western blot detection of the effects of MEK/ERK inhibitor treatment on the expression levels of phosphorylated Fos (p-Fos Ser362) and total Fos protein in cytoplasm and nucleus of uninfected/ILTV-infected cells. HSP90 was used as a cytoplasm-specific internal reference. Lamin B was used as a nucleus-specific internal reference. Tubulin was used as a whole-cell protein loading internal reference. DMSO was used as the solvent control. BI was the Binimetinib inhibitor group. PD was the PD0325901 inhibitor group. (**B**) Indirect immunofluorescence staining combined with laser confocal microscopy observation of the effects of MEK/ERK inhibitor treatment on p-Fos subcellular localization and expression. Upper panel: representative images showing p-Fos (FITC, green), nuclei (DAPI, blue) and merged fluorescence signals. Scale bar, 20 μm. Lower panel: quantitative analysis of the p-Fos nuclear translocation ratio (n ≥ 50 cells per group). * indicates statistically significant difference between groups (*p* < 0.05).

Nuclear enrichment of transcription factors is the core prerequisite for them to initiate transcriptional regulation of downstream target genes. The above studies have confirmed that the MEK/ERK pathway can specifically regulate Fos phosphorylation activation at the post-translational modification level. Based on this, we further dissected the regulatory effect of the MEK/ERK pathway on Fos subcellular localization and nuclear activation, and analyzed the effect of the MEK/ERK pathway on Fos subcellular localization during ILTV infection. We first systematically detected the effect of MEK/ERK inhibitor treatment on Fos phosphorylation level and subcellular distribution by nuclear-cytoplasmic fractionation combined with Western blot. The results showed that in uninfected cells, phosphorylated Fos (p-Fos Ser362) showed only extremely low basal expression in both cytoplasm and nucleus. ILTV infection significantly induced Fos phosphorylation activation, and phosphorylated Fos was mainly enriched in the nucleus, with a significant upregulation of nuclear p-Fos level, while no obvious enrichment of cytoplasmic p-Fos was observed. After treatment with MEK/ERK inhibitors Binimetinib (BI) and PD0325901 (PD), the upregulation effect of nuclear p-Fos induced by ILTV infection was significantly inhibited, while the total Fos protein levels in cytoplasm and nucleus did not change significantly (Figure 3A). Heat shock protein HSP90 was used as a cytoplasm-specific internal reference, lamin protein Lamin B as a nucleus-specific internal reference, and Tubulin as a whole-cell protein loading internal reference, which fully verified the effectiveness of nuclear-cytoplasmic fractionation. To further intuitively verify the above results, we observed the expression and subcellular localization of p-Fos under different treatment conditions by indirect immunofluorescence staining combined with laser confocal microscopy. The results showed that only extremely low green fluorescence signal of p-Fos was detected in uninfected cells. ILTV infection significantly induced upregulation of p-Fos expression, and the green fluorescence signal of p-Fos was highly colocalized with the blue fluorescence signal of DAPI-stained nuclei, indicating that ILTV infection can significantly promote nuclear enrichment of phosphorylated Fos. After treatment with BI and PD inhibitors, the nuclear enrichment effect of p-Fos induced by ILTV infection was significantly inhibited, consistent with the detection results of nuclear-cytoplasmic fractionation Western blot (Figure 3B). We further performed quantitative validation of the immunofluorescence results. For each experimental group, no fewer than 50 cells were randomly selected, and the proportion of cells with predominant nuclear localization of phosphorylated Fos was calculated as the nuclear translocation ratio for intergroup comparison. In mock-infected control cells, the p-Fos nuclear translocation ratio remained at a low basal level. Upon ILTV infection, the ratio in the DMSO vehicle group increased significantly (*p* < 0.05), whereas treatment with either Binimetinib or PD0325901 significantly attenuated this infection-induced nuclear translocation and brought the ratio back to a level close to the uninfected baseline (*p* < 0.05).

These findings further demonstrate that upon ILTV infection, the activated MEK/ERK pathway further directly mediates Fos nuclear translocation by regulating Fos phosphorylation modification, which is the core upstream mechanism for Fos to exert downstream transcriptional regulatory functions.

### 3.4. Fos Functions as the Core Direct Repressing Transcriptional Factor of the Metabolic Genes Repressed by MEK/ERK Pathway

Our previous studies have demonstrated that Fos exerts dual regulatory effects during ILTV replication, which embodies the balanced interplay between this herpesvirus and its hosts. On one hand, Fos directly binds to the promoter of the viral immediate-early gene *ICP4* to positively drive viral gene transcription. On the other hand, Fos modulates host cellular metabolic networks to reshape the energy and biomass supply essential for viral replication, thus indirectly regulating viral proliferation. Given Fos acts as the primary downstream transcription factor within the MEK/ERK signaling cascade, the role of Fos in the repression of host metabolic gene expression by MEK/ERK pathway was addressed.

**Figure 4:**
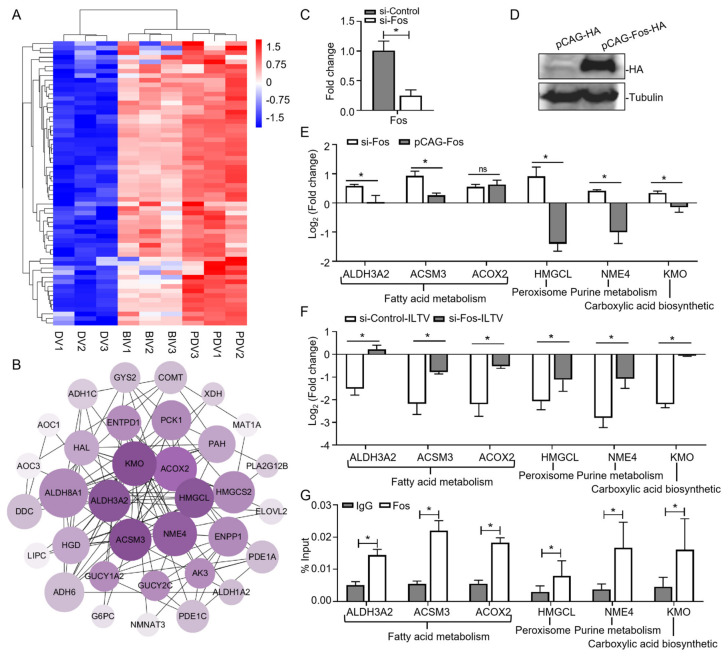
Fos acts as a core direct transcriptional repressor of metabolic genes downstream of the MEK/ERK pathway (**A**) Clustering heatmap showing the expression profile of differentially expressed metabolism-related genes in host cells after treatment with MEK/ERK inhibitors Binimetinib (BI) and PD0325901 (PD). Red indicates upregulated genes, and blue indicates downregulated genes. (**B**) PPI network analysis of differentially expressed metabolism-related genes. Node size is positively correlated with the network centrality (Degree value) of the gene. Node color from dark purple to light purple represents centrality from high to low. (**C**) RT-qPCR detection of Fos-specific siRNA knockdown efficiency. (**D**) Western blot detection of the expression efficiency of HA-tagged Fos overexpression vector. Tubulin was used as the protein loading control. (**E**) RT-qPCR detection of the effects of Fos knockdown/overexpression on the transcription levels of six core metabolic genes. (**F**) RT-qPCR detection of the effects of Fos knockdown on the transcription levels of six core metabolic genes under ILTV infection conditions. (**G**) ChIP-qPCR detection of Fos binding to the promoter regions of six core metabolic genes, with isotype control IgG as the negative control. Data in (**C**,**E**–**G**) are expressed as mean ± SD. *n* = 3. * indicates statistically significant difference between groups (*p* < 0.05). ns indicates no statistically significant difference.

First, we analyzed transcriptome profiles collected from LMH cells treated with the MEK/ERK inhibitor, BI or PD. We then screened genes linked to metabolic processes from the markedly altered metabolic pathways shown in Figure 1E,F before generating a corresponding clustering heatmap. The results showed that specific inhibition of the MEK/ERK pathway in the context of ILTV infection led to significant changes in the transcription levels of genes related to multiple metabolic pathways in host cells. Among them, the transcription levels of core genes in key pathways dependent on ILTV replication, such as fatty acid metabolism, peroxisome, purine metabolism, and carboxylic acid biosynthetic, were significantly upregulated (Figure 4A), which is completely consistent with previous findings that host metabolic pathways are fully activated after MEK inhibition [15,36]. To screen the core target genes of the MEK/ERK pathway regulating host metabolism, we performed PPI network analysis on the above differentially expressed metabolism-related genes. According to node centrality and network connectivity, six key metabolic genes located at the central position of the regulatory network were screened out, namely *ALDH3A2*, *ACSM3* and *ACOX2* in the fatty acid metabolism pathway, *HMGCL* in the peroxisome pathway, *NME4* in the purine metabolism pathway, and *KMO* in the carboxylic acid biosynthetic pathway (Figure 4B).

To verify whether Fos directly regulates the above six core metabolic genes, we first constructed and validated the Fos siRNA interference system and overexpression system. RT-qPCR results showed that siRNA targeting Fos could significantly downregulate the intracellular *Fos* mRNA transcription level, and the knockdown efficiency met the requirements of subsequent experiments (Figure 4C). Western blot results confirmed that the HA-tagged Fos overexpression vector could achieve efficient and stable expression in cells (Figure 4D). Based on the above systems, we detected the regulatory effects of Fos knockdown/overexpression on the transcription levels of the six core metabolic genes by RT-qPCR. The results showed that Fos knockdown significantly upregulated the mRNA transcription levels of *ALDH3A2*, *ACSM3*, *ACOX2*, *HMGCL*, *NME4*, and *KMO*, while Fos overexpression significantly downregulated the transcription levels of these six genes (Figure 4E). Further verification of this regulatory effect under ILTV infection conditions showed that Fos knockdown after ILTV infection also significantly upregulated the transcription of the above six core metabolic genes, consistent with the regulatory trend in the uninfected state (Figure 4F). These results indicate that Fos exerts a direct negative transcriptional regulatory effect on these six core metabolic genes. To clarify whether the negative regulation of Fos on the above metabolic genes is direct transcriptional regulation, we detected the binding of Fos to the promoter regions of the six core metabolic genes by chromatin immunoprecipitation combined with quantitative PCR (ChIP-qPCR). The results showed that compared with the isotype control IgG, Fos was significantly enriched in the promoter regions of *ALDH3A2*, *ACSM3*, *ACOX2*, *HMGCL*, *NME4*, and *KMO* genes (Figure 4G), indicating that Fos can bind to the promoter regions of these metabolic genes and exert negative transcriptional regulation on host core metabolic genes.

In summary, this study confirms that Fos is the core transcription factor downstream of the MEK/ERK pathway regulating host metabolism, and can negatively regulate their transcription by directly binding to the promoters of host core metabolic genes.

### 3.5. MAPK Signaling Pathway Activation and Host Metabolic Reprogramming Are Universal Conserved Features of Alphaherpesvirus Infection

**Figure 5:**
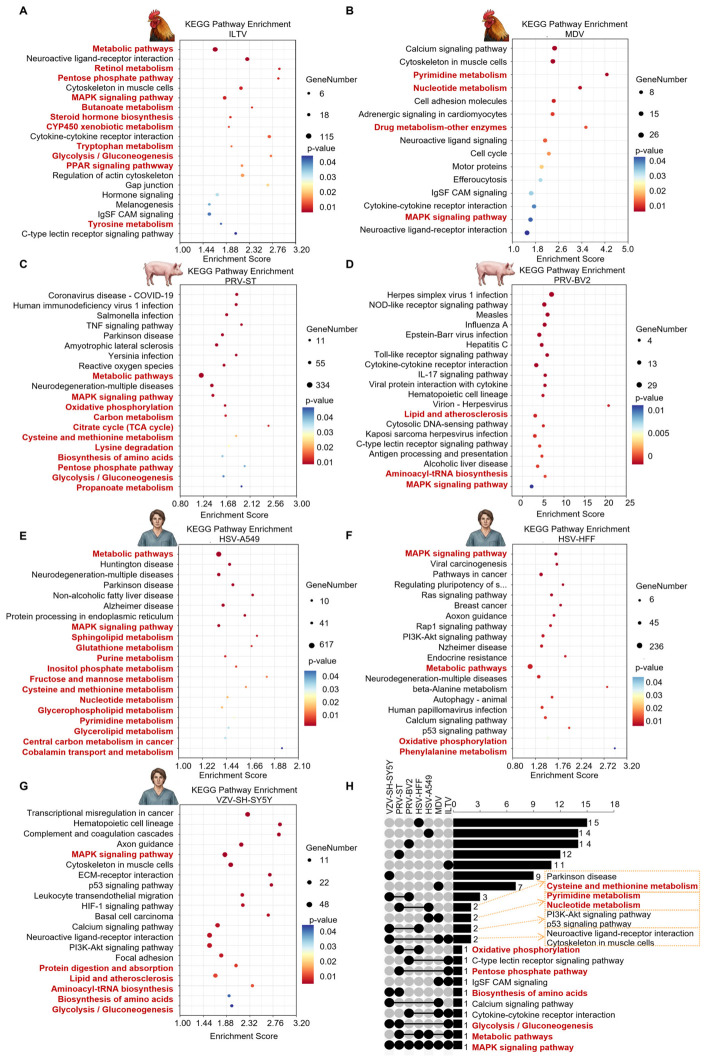
KEGG pathway enrichment analysis of host transcriptomes upon alphaherpesvirus infection (**A**–**G**) KEGG pathway enrichment bubble plots of differentially expressed genes after infection of corresponding host cells with avian infectious laryngotracheitis virus (ILTV), Marek’s disease virus (MDV), pseudorabies virus (PRV, porcine testicular ST cells), pseudorabies virus (PRV, murine microglial BV2 cells), herpes simplex virus type 1 (HSV-1, human lung adenocarcinoma A549 cells), herpes simplex virus type 1 (HSV-1, human foreskin fibroblast HFF cells), and varicella-zoster virus (VZV, human neuroblastoma SH-SY5Y cells), respectively. Bubble size represents the number of differentially expressed genes enriched in the pathway. Bubble color represents the significance *p*-value of pathway enrichment. The MAPK signaling pathway and metabolism-related pathways are labeled in red font. (**H**) Upset intersection analysis plot of significantly enriched pathways in different alphaherpesvirus infection systems, used to show the common and unique characteristics of enriched pathways between different virus infection systems, with core conserved regulatory pathways labeled.

Although alphaherpesviruses vary in host tropism and pathogenicity, their genomic organization, replication strategies, and host dependency are highly conserved [37]. Consistent with previous reports demonstrating that host metabolic reprogramming and MAPK signaling activation are implicated in multiple alphaherpesvirus infections [15,36,38]. Our analyses further reveal that the interplay between MAPK signaling and metabolic regulation is conserved across host species and viral strains during alphaherpesvirus infection. We therefore selected five alphaherpesviruses with distinct host origins, including avian ILTV and Marek’s disease virus (MDV) [26], human herpes simplex virus type 1 (HSV-1) [29,30] and varicella-zoster virus (VZV) [6], and porcine pseudorabies virus (PRV) [27,28], for systematic cross-species and cross-strain comparative analysis. KEGG pathway enrichment analysis showed that after these alphaherpesviruses infected host cells, the host MAPK signaling pathway was significantly enriched in all cases, suggesting that MAPK pathway activation may be a common host response feature during different alphaherpesvirus infections (Figure 5A–G). Meanwhile, metabolism-related pathways are the core response pathways of host cells after infection with these alphaherpesviruses. Multiple metabolic pathways closely related to viral replication, such as glycolysis/gluconeogenesis, nucleotide metabolism, amino acid metabolism, fatty acid metabolism, peroxisome pathway, and PPAR signaling pathway, were significantly enriched in different alphaherpesvirus infection systems, suggesting that host metabolic pathways are highly conserved host response features during alphaherpesvirus infection. To further clarify the core intersection of host metabolic pathways after infection with different alphaherpesviruses, we performed Upset intersection analysis on the significantly enriched pathways after infection with the selected viruses. The results showed that the MAPK signaling pathway is one of the commonly enriched pathways in all tested infection systems. Meanwhile, metabolism-related pathways are the core functional pathways commonly regulated after infection with different alphaherpesviruses, and all tested virus infection systems have consistent regulation of metabolism-related pathways (Figure 5H). The above results suggest that MEK/ERK-related metabolic regulatory characteristics may widely exist in different alphaherpesvirus infection systems.

### 3.6. Fos Serves as a Conserved Core Transcriptional Repressor of Host Metabolic Genes During Alphaherpesvirus Infection

**Figure 6:**
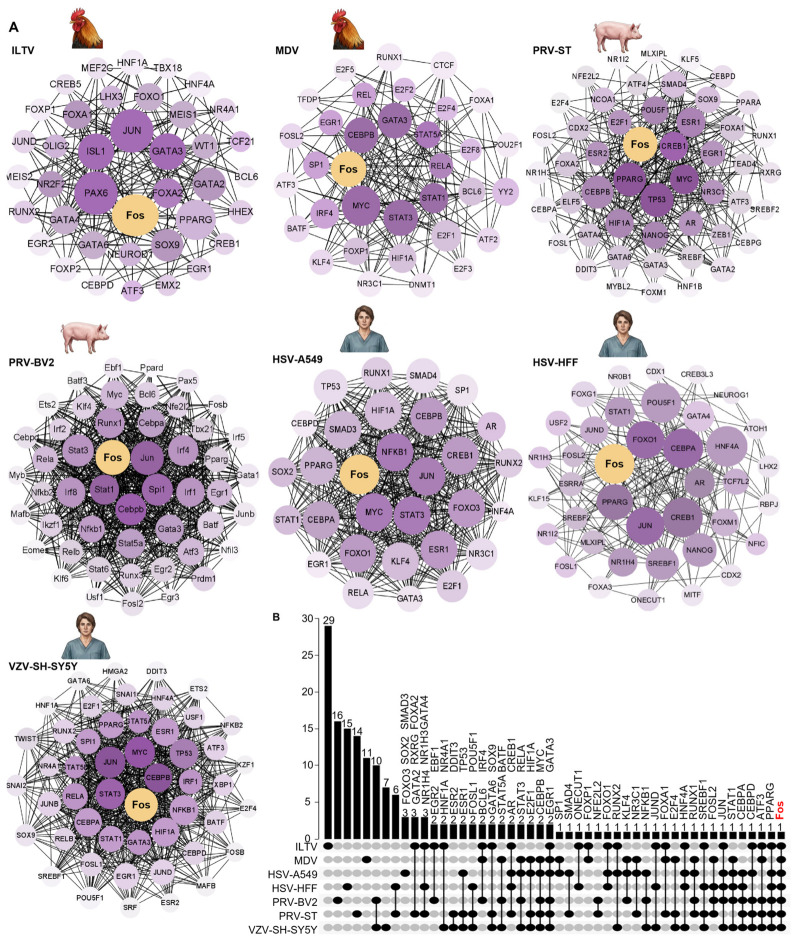
Transcriptional regulatory network analysis of host metabolic pathways during alphaherpesvirus infection (**A**) Protein–protein interaction (PPI) network of upstream transcription factors for metabolism-related differentially expressed genes after infection of host cells with ILTV, MDV, PRV (ST cells), PRV (BV2 cells), HSV-1 (A549 cells), HSV-1 (HFF cells), and VZV (SH-SY5Y cells). Node size is positively correlated with the network centrality (Degree value) of the gene. Node color from dark purple to light purple represents centrality from high to low. The core Hub transcription factor Fos is highlighted in yellow. (**B**) Upset intersection analysis of core upstream transcription factors for metabolism-related differentially expressed genes in different alphaherpesvirus infection systems. The value of the upper bar graph is the number of core upstream transcription factors shared by multiple infection systems in the corresponding intersection combination. Each row in the lower matrix corresponds to an alphaherpesvirus infection treatment group. Each column corresponds to a transcription factor intersection combination. Black solid dots indicate that the treatment group is included in the intersection combination of the current column. Multiple black solid dots in the same column are connected by black lines, indicating that these treatment groups jointly constitute this intersection. The value of the corresponding upper bar graph is the number of core upstream transcription factors shared by these treatment groups.

The above results have confirmed that MAPK signaling pathway activation and host metabolism-related pathways are conserved host response features commonly present during infection with *Alphaherpesvirus* subfamily viruses. However, the upstream core transcriptional regulatory network mediating this cross-species and cross-strain metabolic pathway effect, and whether there is a universal key regulatory node, remain unclear. To address this scientific question, we screened differentially expressed metabolism-related genes after infection for the above five alphaherpesvirus infection systems (avian ILTV, MDV; human HSV-1-infected A549 cells, HFF cells, VZV-infected SH-SY5Y cells; porcine PRV-infected BV2 cells, ST cells), respectively. We performed upstream transcription factor target prediction, constructed transcription factor protein interaction networks, and screened core Hub regulatory genes using Cytoscape software to systematically dissect the conserved transcriptional regulatory mechanism of alphaherpesviruses regulating host metabolic pathways. Transcription factor interaction network analysis showed that in all tested alphaherpesvirus infection systems, Fos had high network connectivity in multiple infection systems, suggesting that it may be involved in the regulation of host metabolic gene expression (Figure 6A). In addition to Fos, transcription factors such as STAT1, CEBPA, CEBPB, ATF3, PPARG, and JUN also occupied core Hub positions in the transcriptional regulatory networks of multiple infection systems, with high network centrality and connectivity, and are conserved auxiliary regulatory factors for alphaherpesviruses regulating host metabolic gene expression. Among them, in five infection systems including ILTV, HSV-1 (A549, HFF), PRV-BV2, and VZV-SH-SY5Y, JUN and Fos were jointly located at the core position of the transcriptional regulatory network, serving as core upstream regulatory factors for metabolism-related genes. We further performed intersection analysis on the core transcriptional regulatory factors of all systems. The results showed that Fos is one of the upstream transcription factors commonly predicted in all tested infection systems, and is the most conserved core regulatory node for alphaherpesvirus-mediated host metabolic pathway reprogramming. JUN, STAT1, CEBPA, CEBPB, ATF3, and PPARG are conserved auxiliary core factors for metabolic regulation common to most alphaherpesvirus infection systems, together constituting the conserved transcriptional regulatory network for alphaherpesviruses regulating host metabolic gene expression (Figure 6B).

The above results are fully consistent with the MEK/ERK-Fos metabolic regulation we elucidated in ILTV. Fos and JUN are core members of the activator protein 1 (AP-1) transcription factor family. Fos needs to form a high-affinity heterodimer with JUN family proteins through the C-terminal leucine zipper motif to specifically bind to the TRE cis-acting element in the target gene promoter and exert transcriptional regulatory function [39]. The MAPK/ERK pathway is the most core upstream regulatory pathway for AP-1 complex activity. ERK can directly phosphorylate the conserved serine site of Fos to enhance Fos protein stability and transcriptional activity. Combined with the ILTV experimental results and cross-virus transcriptome analysis, it is suggested that the MAPK/Fos-related regulatory mode may have certain conservation during different alphaherpesvirus infection processes.

**Figure 7:**
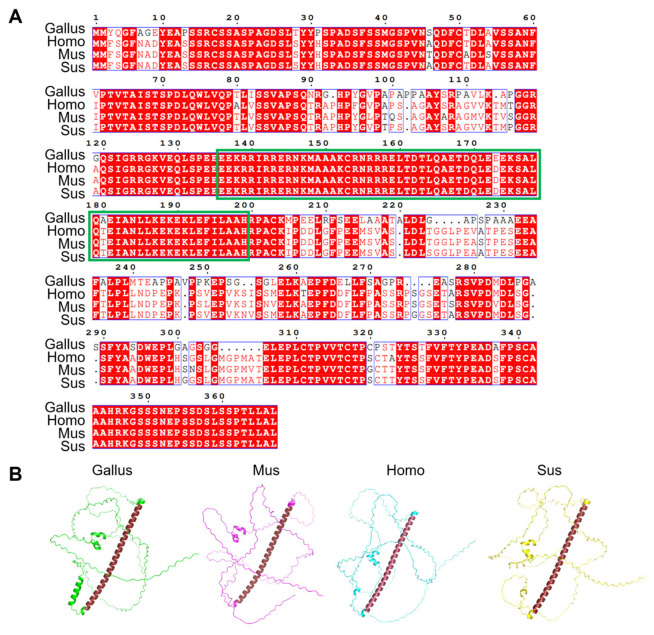
Cross-species sequence conservation and three-dimensional structural analysis of Fos proteins (**A**) Multiple sequence alignment results of full-length amino acid sequences of Fos proteins from four species: chicken (*Gallus gallus*), mouse (*Mus musculus*), human (*Homo sapiens*), and pig (*Sus scrofa*), completed using the EMBL-EBI online MUSCLE tool. Completely conserved identical amino acid residues are labeled in red. Physicochemically similar amino acid residues are labeled in blue. The Fos protein DNA-binding domain (DBD) is indicated by the green box. (**B**) Three-dimensional structure homology modeling results of chicken, mouse, human, and porcine Fos proteins, completed by AlphaFold3 and visualized by PyMOL software. The brown α-helix region is the Fos DNA-binding domain.

Having established Fos as the core downstream effector of the MEK/ERK pathway in ILTV infection, we next investigated whether this regulatory role is conserved across alphaherpesvirus host species. To elucidate the molecular basis of its potential universal regulatory function, we systematically analyzed the sequence and structural conservation of Fos proteins from four representative natural hosts of alphaherpesviruses: chicken (*Gallus gallus*), mouse (*Mus musculus*), human (*Homo sapiens*), and pig (*Sus scrofa*). Multiple sequence alignment results showed that the full-length sequences of Fos proteins from chicken, mouse, human, and pig are highly evolutionarily conserved (Figure 7A). Among them, completely conserved amino acid residues labeled in red are widely distributed throughout the protein, and core functional regions show continuous complete conservation. Physicochemically similar residues labeled in blue account for an extremely high proportion of non-completely conserved sites, further maintaining the overall functional conservation of the protein. The Fos DNA-binding domain (DBD, bZIP leucine zipper core functional region) labeled in the green box is almost completely conserved in amino acid residues among the four species. The C-terminal bZIP leucine zipper domain (corresponding to the sequence 130~200 aa region), which is the core functional region for Fos to form AP-1 heterodimers with JUN and bind to the TRE cis-acting element in the target gene promoter, has an amino acid sequence identity of more than 96% among the four species and 100% identity among the three mammals [39]. Meanwhile, the core site of MEK/ERK-mediated phosphorylation modification identified in our previous study (chicken Fos Ser349, corresponding to human Fos Ser362) and its surrounding MAPK recognition motif AAHRKGSSNE are completely conserved among the four species, suggesting that this phosphorylation-related motif is highly conserved across different species.

Three-dimensional structure homology modeling analysis showed that the overall spatial conformations of Fos proteins from the four species are highly conserved (Figure 7B). The main body of the protein is a long and regular α-helix structure, among which the α-helix structure labeled in brown is the core bZIP leucine zipper functional domain. The length, folding mode, and spatial orientation of this helix are completely consistent among the four species, which is the core structural basis for its dimerization and DNA-binding functions. The remaining regions at the N-terminus and C-terminus of the protein are intrinsically disordered regions (IDRs) without fixed secondary structure. The structural differences of Fos proteins among the four species only exist in the flexible conformations of non-core disordered regions. Among them, the N-terminal disordered region of chicken Fos has a short auxiliary helix, which is different from the N-terminal conformations of mouse, human, and porcine Fos. However, this region does not participate in the core functions of Fos-JUN dimerization, DNA binding, and transcriptional activation. The core functional helix and the spatial exposure characteristics of phosphorylation sites are completely conserved among the four species. The target phosphorylated serine sites of all species are located in the C-terminal disordered region, completely exposed on the protein surface without secondary structure wrapping, and have sufficient kinase modification accessibility (Figure 7B).

Collectively, systematic bioinformatics analyses in this study demonstrate that the core functional domains and key phosphorylation sites of Fos protein exhibit high conservation across diverse host species of alphaherpesviruses. This provides important sequence and structural evidence for the potential conservation of MAPK/Fos-mediated regulatory patterns in different hosts and lays a foundation for further refining the theoretical framework of conserved molecular mechanisms underlying alphaherpesvirus-host interactions.

## 4. Discussion

*Alphaherpesvirus* subfamily viruses can infect multiple host species such as humans, pigs, and poultry, causing serious zoonotic diseases and economic losses in the livestock and poultry industry [40]. Virus–host crosstalk of this virus subfamily has long been a major virology research focus. Our previous work has demonstrated that host MAPK signaling pathway activation and cellular metabolic network are core host response events during alphaherpesvirus infection. Within this signaling network, the MEK/ERK pathway, as one of the key cascade reactions of the MAPK family, plays an important role in host antiviral defense [15,36]. Meanwhile, work from our group has further shown that the AP-1 family transcription factor Fos is a key host factor for avian alphaherpesvirus ILTV infection, and can bidirectionally regulate ILTV replication by directly targeting the viral immediate early gene *ICP4* and host metabolic key enzyme-encoding genes [17,22]. However, it remained unclear how MEK/ERK precisely rewires host metabolism via downstream transcription factors, and whether this regulatory axis is conserved across alphaherpesvirus species and strains. Here, using ILTV as a model, we systematically uncovered the full molecular cascade whereby MEK/ERK governs infection-triggered metabolic reprogramming through Fos. We further verified Fos as an evolutionarily conserved master transcription factor controlling host metabolism across alphaherpesviruses, offering novel theoretical support for developing broad-spectrum antiviral targets.

We characterized dual transcriptional and post-translational control of Fos by MEK/ERK. ILTV infection strongly activates MEK/ERK, whereas MEK/ERK inhibitors abolish infection-induced Fos upregulation and phosphorylation. By contrast, MEK/ERK blockade barely alters JUN phosphorylation, demonstrating selective regulation of Fos rather than other AP-1 members. This result confirms the specificity of MEK/ERK pathway regulation on Fos. This finding is consistent with our previous research results. Previous studies showed that in the ILTV infection model, Fos knockdown can significantly inhibit ILTV replication, while JUN knockdown has no significant effect on ILTV gene transcription and progeny virus production [17]. Phosphorylation site alignment and structural modeling pinpointed chicken Fos Ser349 (human ortholog Ser362) as the key MEK/ERK modification site. This kinase-recognition motif is fully conserved across species, resides within an intrinsically disordered protein region and is surface-exposed, enabling efficient post-translational modification by MEK/ERK [39].

Transcription factors require nuclear translocation to execute transcriptional functions. Combined nuclear-cytoplasmic fractionation and confocal imaging showed that ILTV drives robust nuclear accumulation of phosphorylated Fos, an effect fully reversed by MEK/ERK inhibition, without altering total Fos subcellular distribution. This demonstrates that MEK/ERK regulates Fos function primarily through phosphorylation-dependent nuclear enrichment, not total protein abundance. Mammalian studies have established that ERK-mediated Fos Ser362 phosphorylation stabilizes Fos and retains it in the nucleus to boost transcriptional activity. We provide the first validation of this conserved regulatory mode in avian cells and confirm that nuclear translocation of phosphorylated Fos is required for subsequent repression of metabolic genes.

Having delineated the upstream regulatory mechanism of Fos, we identified Fos as a core metabolic effector downstream of the MEK/ERK pathway. Transcriptomic analysis revealed that MEK/ERK inhibition reverses ILTV-induced metabolic suppression and upregulates core genes supporting viral replication across fatty acid, peroxisomal and purine metabolism pathways. Consistent with our recent findings, MEK/ERK activation restricts ILTV propagation via broad metabolic suppression, whereas pathway inhibition relieves this metabolic block and provides energy and substrates to support viral replication. Through protein–protein interaction (PPI) network analysis, we further screened six core metabolic genes within this regulatory network, namely *ALDH3A2*, *ACSM3*, *ACOX2*, *HMGCL*, *NME4* and *KMO*. Combined overexpression, knockdown and ChIP-qPCR assays confirmed that Fos directly binds to and represses the promoters of all six genes, providing robust evidence for Fos-mediated transcriptional repression of these targets. Subsequent work will include luciferase reporter assays using representative promoter fragments harboring AP-1 binding motifs to further delineate the precise binding and regulatory mechanism.

Notably, all six genes encode core catalytic components of the fatty acid, peroxisomal and purine metabolic pathways, which our prior untargeted metabolomic work has validated as essential for efficient ILTV replication. Their expression levels directly govern the metabolic flux of the corresponding pathways, and Fos-mediated transcriptional repression of these genes is highly consistent with earlier metabolomic observations, forming a complete evidence chain ranging from metabolic enzyme expression and metabolite abundance to viral replication phenotypes. We will perform gain-of-function rescue of representative metabolic genes in Fos-overexpressing cells, combined with viral replication assays and targeted metabolomic profiling. These experiments will clarify the role of these metabolic genes in Fos-mediated antiviral effects and further dissect the dose-dependent regulatory pattern and underlying molecular details of this regulatory axis.

Our prior work showed that Fos controls the TCA cycle and ATP synthesis via *MDH1* and *ATP5A1*. In this study, we extend the metabolic regulatory network of Fos to multiple key pathways essential for ILTV replication, including fatty acid metabolism, peroxisomal metabolism, carboxylic acid biosynthetic and purine metabolism, and fully delineate the core molecular mechanism underlying the antiviral function of the MEK/ERK pathway. Activated upon ILTV infection, the MEK/ERK pathway promotes nuclear translocation and activation of its downstream transcription factor Fos, which directly targets the promoters of core genes across multiple metabolic pathways and represses their transcription. This regulatory action broadly suppresses host metabolic processes required for viral replication and ultimately mediates host antiviral defense. The bidirectional effect of Fos on ILTV replication has been systematically verified in our prior studies using the same LMH-ILTV infection model. Fos can either promote viral transcription by directly binding to the promoter of the viral immediate early gene *ICP4* or modulate the supply of biomolecules and energy for viral proliferation by reshaping host metabolic networks [17,36,41]. This work specifically focuses on the MEK/ERK-dependent metabolic repressive branch of Fos function, a host defense arm distinct from its direct proviral transcriptional activity.

The MEK/ERK pathway is a core signaling cascade governing fundamental cellular physiological processes. Direct genetic knockdown of MEK1/2 or ERK1/2 often results in severe disruption of cellular homeostasis and even cell death, representing a well-recognized technical limitation in the field. Accordingly, highly selective small-molecule inhibitors represent a prevailing and widely accepted strategy for dissecting MEK/ERK functions and have been extensively adopted in alphaherpesvirus research. For instance, MEK-specific inhibitors were employed to uncover the phase-dependent functional divergence of the ERK pathway during herpes simplex virus type 1 (HSV-1) infection [12], and the MEK inhibitor was used to demonstrate that the Ras/MEK/MAPK-c-Fos axis regulates the initiation of herpes simplex virus type 2 (HSV-2) replication [14]. In this study, we selected two structurally distinct, highly selective MEK1/2 inhibitors, Binimetinib (BI) and PD0325901 (PD). Our prior validation confirmed that at the working concentrations used, both inhibitors achieve over 90% inhibition of ERK1/2 phosphorylation without cross-inhibition of the p38 MAPK or JNK pathways, effectively ruling out off-target effects [15]. We employed a strategy combining pharmacological inhibition with genetic validation of downstream effectors to dissect this regulatory cascade. Genetic manipulations of Fos further validated its regulatory effects on the identified metabolic targets, providing downstream genetic evidence to support the antiviral role of the MEK/ERK pathway. Given that Fos exerts bidirectional effects on ILTV replication by directly regulating viral gene transcription as well as modulating host metabolic networks, functional rescue assays under MEK/ERK inhibition are needed to rigorously confirm the causal link between MEK/ERK-driven Fos nuclear translocation and the observed metabolic and antiviral phenotypes. We will carry out these experiments in follow-up work.

Furthermore, the MAPK family is a highly conserved signaling network comprising canonical branches such as ERK, p38, and JNK. Its effects during viral infection are highly context-dependent, shaped collectively by pathway specificity, infection phase, and host cell type [10,11]. The p38 and JNK branches are frequently hijacked by viruses to support viral gene transcription and replication, whereas the ERK branch exhibits more diverse functions that shift dynamically across infection stages [7,15]. The present study focuses on the middle-to-late stages of ILTV infection, which explains the predominant antiviral phenotype of the MEK/ERK-Fos metabolic axis.

Although *Alphaherpesvirus* subfamily viruses have significant differences in host range and pathogenicity, their genome structure, replication cycle, and host interaction mode are highly evolutionarily conserved. Developing cross-species broad-spectrum prevention and control targets has always been the core research goal in this field. Transcriptomic profiling of five alphaherpesviruses (ILTV, MDV, HSV-1, VZV, PRV) across seven infection systems revealed MAPK activation as the only universally enriched host signaling response, with metabolic remodeling as another conserved infection signature. Among upstream transcription factors governing metabolism-related DEGs, Fos is the sole shared hub across all tested systems, while JUN, STAT1 and CEBPA only exert auxiliary roles in partial models. This finding extends the MEK/ERK pathway activation of Fos to regulate host cell metabolic network, which we elucidated in ILTV, to the entire *Alphaherpesvirus* subfamily. We will validate the cross-species conservation of this cascade in HSV-1, VZV, PRV and MDV via combined MEK/ERK intervention and Fos rescue assays in future work.

To investigate the conserved regulatory role of Fos across species, we performed multi-sequence alignment and homology modeling of Fos proteins from chicken, mouse, human and pig. The core bZIP leucine zipper DNA-binding domain shares more than 96% sequence identity across all four species, and the MEK/ERK phosphorylation motif is fully conserved. The kinase recognition motif, where the core site of MEK/ERK-mediated phosphorylation modification is located, is completely conserved among the four species. Three-dimensional structure analysis confirmed that the core functional helix conformation and the spatial exposure characteristics of phosphorylation sites of Fos proteins from the four species are completely consistent, with only flexible conformation differences in non-core disordered regions. Sequence and structural conservation provide a molecular basis for Fos to mediate universal metabolic regulation across alphaherpesvirus hosts. Meanwhile, we previously reported a direct protein interaction between Fos and p53, and the two can jointly target the ILTV *ICP4* promoter to regulate viral transcription. Recent studies have confirmed that chicken p53 can exert broad-spectrum antiviral effects by regulating host metabolism, and Fos is a core synergistic transcription factor in the p53 metabolic regulatory network. This implies the Fos-p53 complex acts as a central hub coordinating metabolism and immunity during alphaherpesvirus infection, representing a promising direction for future research.

This work defines the mechanism by which the MEK/ERK pathway mediates host defense against ILTV through Fos-driven metabolic repression, providing a new host-targeted strategy for alphaherpesvirus intervention. Host-directed antiviral approaches carry a lower risk of viral drug resistance, and the cross-species conservation of Fos further highlights its potential as a broad-spectrum antiviral target. MEK inhibitors are already well established in clinical oncology with well-characterized safety and pharmacokinetic profiles, offering a solid foundation for drug repurposing and future evaluation as adjuvant antiviral agents in poultry production. We are currently developing chicken embryo and chick infection models to validate this regulatory axis in vivo, which will provide further translational evidence for this mechanism.

In summary, this study systematically elucidated the molecular mechanism by which the MEK/ERK pathway mediates host metabolic gene changes through the downstream transcription factor Fos, and confirmed for the first time that Fos is a cross-species conserved core transcription factor regulating host metabolism by *Alphaherpesvirus* subfamily viruses, laying a solid theoretical foundation for the development of broad-spectrum alphaherpesvirus prevention and control targets.

## Figures and Tables

**Table 1:** Primers used for plasmid construction, viral specific qPCR, and RT-qPCR.

Primer Name	Sequence (5′-3′)
*Fos*	GTGAGAGCTGGTAGTCTGT
	ATATTGCCAGGAACACAGTAG
*Jun*	CAGCATCACATAAACCCCCAG
	TCATGCGTTTTCTCTCGGCTT
*β-actin*	GTGGATCAGCAAGCAGGAGT
	ATAAAGCCATGCCAATCTCGT
*ILTV-gC*	AAATGCTACGACCTGAAACT
	CTCGGGCTCATCCAAAACA
*Fos*	GGGGTACCATGTACCAGGGCTTCGCTGGGG
	CCGCTCGAGCAAGGCCAGCAGGGTGGGGGAGC
*ALDH3A2*	TTTCTTCCTCGCATCCTTAC
	TGTTCGTGCCCTCCATCT
*HMGCL*	GTGACACCATTGGCATCG
	CACCAGGTCCTCTGTAGCG
*KMO*	CTGAGTTCACCAGGCTGAGG
	GTCGAATTGACGTGCTCTCG
*ACSM3*	CACCCCGTGGCCATCTATTT
	ATGTGCAGCTTCCACTCACT
*NME4*	CATGGTGGGGGATACGGATT
	TCCCTTTGGAACCAGAAGCC
ACOX2	TTGGAAGGAGCTGTGCGATA
	CTTGGTGGTGTCCAGTAGGG
C-ALDH3A2	TAGTAACGCCTTGGAATC
	AGCCTGCAACATACAAAA
*C-HMGCL*	ACAGTCTGAGGCAAGGAG
	TTTGGACCCATCAGTAAG
*C-KMO*	GCTGGAAGTTAATGCTGT
	AGAGGGTAGAATGTGAGGTA
*C-ACSM3*	ACACTGCTGCCTCACGTC
	TATGGGCTGGGAGAAGAA
*C-NME4*	TGCCTATGGAGTCGTCTTG
	CGAAGGTACGAATGGCTGA
*C-ACOX2*	CTAACCCATTCCCCAGTC
	TGTGGCTCAGATGCTTTAC

Note: The restriction enzyme sites in primer sequences are underlined. Primer names prefixed with ‘C-’ denote ChIP-qPCR primers; all others are RT-qPCR primers.

## Data Availability

The raw RNA sequencing data generated in this study have been deposited in the NCBI BioProject under accession number PRJNA1079397. All other data supporting the findings of this study are available within the manuscript.
